# Assessment of health-related quality of life in patients with cystinuria on tiopronin therapy

**DOI:** 10.1007/s00240-019-01174-6

**Published:** 2019-12-13

**Authors:** Frank Modersitzki, David S. Goldfarb, Ross L. Goldstein, Roger L. Sur, Kristina L. Penniston

**Affiliations:** 1 Department of Medicine, New York University School of Medicine, NYU Langone Health, 550 First Avenue, OBV A600, New York, 10016 USA; 2 Retrophin, Inc., San Diego, CA USA; 3 University of California San Diego School of Medicine, San Diego, CA USA; 4 University of Wisconsin School of Medicine and Public Health, Madison, WI USA

**Keywords:** Heath-related quality of life, Cystinuria, Kidney stones, Tiopronin, Urolithiasis

## Abstract

Cystinuria comprises less than 1% of kidney stones and is associated with impaired health-related quality of life (HRQOL). Limited evidence is available regarding HRQOL of patients with cystinuria treated with tiopronin (Thiola^®^). The objective of this study was to assess the HRQOL of patients with or without tiopronin treatment. For this cross-sectional survey, patients on tiopronin treatment were recruited through the “Thiola^®^ Total Care Hub,” a specialty pharmacy used to dispense tiopronin, and compared with patients not taking tiopronin (non-tiopronin group) who were identified from the Cystinuria Contact Registry at New York University School of Medicine. Consented patients responded to a survey that included questions about their experiences with kidney stones, the Wisconsin stone quality of life (WISQOL) (disease-specific) questionnaire, and the short form-36 version 2 (SF-36v2) (generic) HRQOL questionnaire. Statistical analyses included independent-sample *t* tests, one-way analysis of variance (ANOVA), and correlations. The survey was completed by 312 patients: 267 in the tiopronin group (144 male, 123 female; mean 49 years) and 45 in the non-tiopronin group (10 male, 35 female; mean 48 years). Both groups utilized pain medications similarly (24% overall). Patients on tiopronin had a significantly better HRQOL than patients not on tiopronin for all WISQOL domains (*p *< 0.001) and all but the physical functioning SF-36v2 domain (*p *< 0.001), where both groups approached the US normative mean, when controlling for the last stone event. Compared with patients in the non-tiopronin group, patients taking tiopronin reported better HRQOL on both the WISQOL and SF-36v2.

## Introduction

Cystinuria is a rare genetic disorder characterized by an impaired transport of cystine and dibasic amino acids across the luminal membrane of the renal proximal tubule and small intestine. Increased urinary cystine concentrations lead to formation of recurrent renal stones [1]. Cystinuria accounts for about 1% of all kidney stones [2]. More than 80% of cystinuria patients will experience their first symptomatic stone in the first 2 decades of life and then often have recurrent stones every 3–5 years or more frequently [3, 4]. Possible complications of cystinuria include renal colic, hematuria, obstruction, and infections of the urinary tract [3]. Urological intervention is often required for recurrent and larger stone formation episodes. Recurrent episodes also have a significant impact on patients’ daily and professional activities, and on emotional and mental health [5]. Patients with recurrent stones have increased stress and depression [6–8].

Cystinuria affects 1/10,000 people in the United States (US) and 1/7000 worldwide [9]. Standard management involves prescription of adequate oral fluid intake, low sodium and protein diet, urinary alkalinization, and cystine-binding thiol medications to decrease urinary cystine below 250 mg/L to increase solubility.

Tiopronin (Thiola^®^, Mission Pharmacal Company, San Antonio, TX) was approved by US FDA in 1988 for treatment of cystinuria. Tiopronin is an active reducing agent that undergoes thiol-disulfide exchange with cystine to form a mixed disulfide of water-soluble tiopronin-cysteine, thus reducing the less-soluble urinary cystine [10, 11]. In a clinical trial, tiopronin prevented stone formation and reduced the rate of new stone formation in patients previously treated with d-penicillamine (71.4% and 94.1%, respectively) and not previously treated with d-penicillamine (62.8% and 81.4%, respectively) [12]. The most common adverse events associated with tiopronin are nausea, diarrhea or soft stools, oral ulcers, rash, fatigue, fever, arthralgia, proteinuria, and emesis [11].

In addition to the endpoints used in cystinuria clinical trials (stone formation, cystine excretion, frequency of surgical intervention), it is equally important to consider patients’ health-related quality of life (HRQOL) and the impact treatment has on HRQOL. HRQOL is multidimensional and includes psychosocial, physical, and emotional status, as well as patient autonomy [13]. HRQOL is an individual’s perception of his or her health and health-related aspects [14].

Relatively few studies have evaluated HRQOL in kidney stone formers or in people with cystinuria. The two studies that have been conducted indicate that patients with cystine stones have lower HRQOL compared with the US standard population and non-cystine stone formers [15, 16].

Cystinuria patients treated with tiopronin are hypothesized to have fewer HRQOL decrements compared with patients not receiving tiopronin treatment. The objective of this study was to assess HRQOL in a large cystinuria patient population using the disease-specific Wisconsin stone quality of life (WISQOL) and general MOS short form-36 version 2 (SF-36v2) questionnaires [17].

## Materials and methods

### Study design

This study was a cross-sectional survey designed to assess HRQOL in cystinuria patients enrolled in the Cystinuria Contact Registry at New York University (NYU) School of Medicine and in cystinuria patients included in the “Thiola^®^ Total Care Hub,” a specialty pharmacy used to dispense tiopronin; surveys were mailed out to members of both registries. The study was approved by the Western Institutional Review Board (Western IRB 1172206 IRB Protocol# 20170185). Data collection, raw data storage, and data handling were conducted by a third-party Health Insurance Portability and Accountability Act (HIPAA)-compliant data management vendor. The study was sponsored by Retrophin, Inc.

### Study population

Participants taking tiopronin treatment were recruited through the Total Care Hub pharmacy. All participants in the Total Care Hub who matched the inclusion criteria were offered participation in the study. Participants were included if they were cystinuria kidney stone formers, were on stable tiopronin treatment, were ≥ 18 years of age, had a valid home address, and could speak English. Cystinuria patients not taking tiopronin were identified from the Cystinuria Contact Registry at NYU School of Medicine. Those ≥ 18 years of age, living in the US, and who agreed to be contacted for a clinical trial were included in the study as a control group. As stated in the consent form, all participants understood that the Total Care Hub and this research project were sponsored by Retrophin, Inc. and that participation in the trial was not obligatory.

### Data collection

All participants who consented to participate were provided SF-36v2 and the WISQOL questionnaires. Information about participants’ underlying stone disease and treatment was also collected. All questionnaires were administered via mail. Data were transferred into IBM^©^ SPSS Statistics, version 22, for statistical analysis and storage.

### Statistical analysis

All survey data were collected by a HIPAA-compliant third-party data management vendor. De-identified patient data were then provided to Retrophin, Inc. for analysis. Current dose (mg) and prescription variables (taking tiopronin as prescribed: yes, no, not taking) were used to assign participants to the tiopronin or control groups. Participants taking tiopronin as prescribed were included in the tiopronin group and those without a prescription and not taking tiopronin were included in the control group. Descriptive statistics on demographics, morbidity variables, treatment history, and HRQOL scores were used to compare results between the treatment groups. Based on the type of data, Chi-squared, univariate analysis of variance (ANOVA), Mann–Whitney *U*, or Student’s *t* test were used. Time between the last stone event and HRQOL assessment was accounted for; responses were grouped based on the time between completion of the survey and the last stone event (≤ 30 days, 31–365 days, and > 365 days). One-way ANOVA was used to calculate differences within and between HRQOL domains [16]. For all statistical tests, *p* ≤ 0.05 was considered to be significant. Multiple regression analyses included the following variables: age, gender, time since last stone event, comorbidities, and tiopronin group (on treatment and not on treatment). Cronbach’s alpha and intraclass correlation was used to determine the internal consistency of the SF-36v2 and WISQOL. Correlations were interpreted as poor (≤ 0.20), fair (0.21–0.40), moderate (0.41–0.60), good (0.61–0.80), and excellent (> 0.80). Cronbach’s alpha coefficients were interpreted as unacceptable (< 0.50), poor (0.50–0.60), questionable (0.61–0.70), acceptable (0.71–80), good (0.81–0.90), and excellent (> 0.90).

## Results

### Subject demographics

A total of 312 participants were enrolled in the study, 267 (85.6%) on tiopronin treatment and 45 (14.4%) not on tiopronin treatment (Table 1). The overall response rate for patients opting to participate was 23% (patients on tiopronin, 267/1113 [24%]; patients not on tiopronin, 45/264 [17%]). The proportions of female (46.1%) and male (53.9%) participants were comparable in the tiopronin group, while there were more females (78.8%) in the non-tiopronin group. The mean number of events, mean number of events requiring hospitalization, mean number of events requiring surgery, and mean number of extracorporeal shock wave lithotripsies were all higher in the tiopronin group.

**Table 1:** Baseline characteristics

	Tiopronin	Non-tiopronin	*p* value^a^
Number (%) of patients	267 (85.6)	45 (14.4)	–
Age, years			
Mean (SD)	49.12 (15.89)	48.42 (14.70)	0.784
Median (range)	51 (18–88)	47 (19–76)	0.680
Sex, *n* (%)			
Female	123 (46.1)	35 (78.8)	< 0.001
Male	144 (53.9)	10 (22.2)	
Age of first stone, years			
Mean (SD)	20.5 (11.7)	17.0 (9.9)	0.068
Median (range)	18 (2–66)	15 (2–43)	0.032
Total number of events			
Mean (SD)	35.3 (93.2)	22.6 (36.2)	0.561
Median (range)	10 (0–625)	9 (1–150)	0.763
Number of events requiring hospitalization			
Mean (SD)	10.6 (32.8)	6.0 (9.5)	0.464
Median (range)	4 (0–300)	2.5 (0–40)	0.147
Number of events requiring surgery			
Mean (SD)	11.3 (53.8)	6.9 (10.1)	0.656
Median (range)	4 (0–740)	3.5 (0–50)	0.459
Extracorporeal shock wave lithotripsy (SWL)			
Prevalence of SWL procedures, *n* (%)	109 (41)	17 (38)	0.796
Median (IQR)	2.0 (4)	2.0 (4)	0.828
Taking tiopronin as prescribed, *n* (%)	254 (95.1)	NA	NA
Missed doses of tiopronin, *n* (%)			
Rarely	125 (46.8)	NA	NA
Daily	52 (19.5)	NA	NA
Taking any pain medication, *n* (%)	28 (10.5)	7 (15.6)	0.319
Taking daily prescription pain medication, *n* (%)	63 (23.6)	16 (35.6)	0.389
Missed daily doses of alkalinizing medications, *n* (%)	11 (4.1)	1 (2.2)	0.051

*IQR* interquartile range, *NA* not applicable

^a^Independent *t* tests were used for mean values; nonparametric Mann–Whitney *U* test was used for medians

High proportions of patients in both the tiopronin and non-tiopronin groups were taking any pain medication (tiopronin: 10.5%; non-tiopronin: 15.6%) and prescription pain medication on a daily basis (tiopronin: 23.6%; non-tiopronin: 35.6%). The majority of the patients in the tiopronin group (95.1%) reported taking tiopronin as prescribed; however, 46.8% of patients reported rarely missing doses and 19.5% reported missing doses on a daily basis.

Time between first stone and cystinuria diagnosis was 4 years for patients in the tiopronin group and 1 year for those in the non-tiopronin group.

There was no statistical difference between the tiopronin and non-tiopronin groups for prevalence of in 9 of 13 comorbidities (Table 2). Statistically significant differences in comorbidity prevalence between the tiopronin and non-tiopronin groups were found for musculoskeletal disease, endocrine (e.g., thyroid) disorder, neurologic disorder, and skin disorder.

**Table 2:** Comorbidities

	Tiopronin *n* (%)	Non-tiopronin *n* (%)	Total *n* (%)	*p* value
Chronic anemia	19 (7.3)	7 (15.6)	26 (8.5)	0.067
Malignancy	26 (10.0)	7 (15.6)	33 (10.8)	0.264
Coronary artery or cardiovascular disease	71 (26.9)	11 (24.4)	82 (26.5)	0.861
Pulmonary (respiratory) disease	27 (10.3)	4 (8.9)	31 (10.1)	0.777
Musculoskeletal disease^a^	51 (19.4)	16 (35.6)	67 (21.8)	0.049
Diabetes	28 (10.6)	7 (15.9)	35 (11.4)	0.309
Gastrointestinal tract disease	27 (10.3)	8 (18.2)	35 (11.4)	0.126
Endocrine (e.g., thyroid) disorder^a^	14 (5.3)	9 (20.5)	23 (7.5)	< 0.001
Neurologic disorder^a^	16 (6.1)	11 (25.0)	27 (8.8)	< 0.001
Anxiety	83 (31.4)	17 (38.6)	100 (32.1)	0.345
Depression	80 (30.4)	16 (35.6)	96 (31.2)	0.492
Psychiatric disorder (other than anxiety or depression)	14 (5.3)	2 (4.5)	16 (5.2)	0.834
Skin disorder^a^	27 (10.2)	11 (24.4)	38 (12.3)	0.007

^a^Pearson Chi-squared test *p* < 0.05

### HRQOL scores

#### SF-36v2 scores

**Fig. 1:**
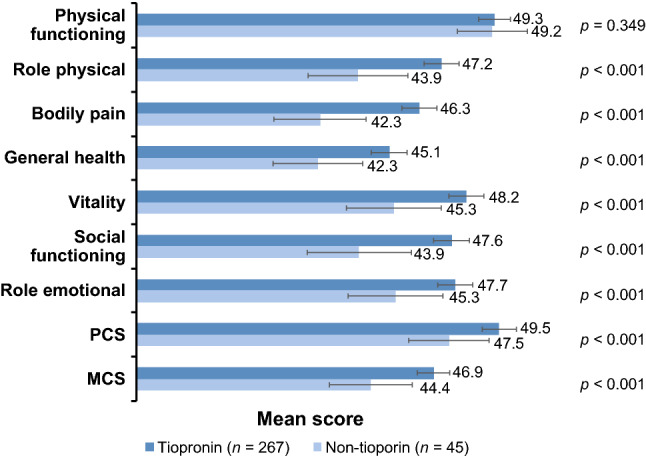
Mean SF-36v2 domain scores by treatment group. Error bars indicate standard error of the mean. *p* values for tiopronin vs non-tiopronin groups (ANOVA) for each domain when controlled for time since the last stone event. *MCS* mental component scores, *PCS* physical component scores

Tiopronin- and non-tiopronin-treated patients had SF-36v2 scores < 50 (the normative US mean). All HRQOL domains except for physical functioning were higher for the tiopronin group compared with the non-tiopronin group (Fig. 1) when controlling for time since the last stone event. When using a generic HRQOL instrument, it is important to control for the time between the last stone event and the survey. Twenty-two of the 36 questions of SF-36v2 are asking about a participant’s quality of life for the previous 30 days. The Levene test of variance revealed that in all domains except physical functioning, the variances were not equal.

Physical function was 49.3 for the tiopronin group and 49.2 for the non-tiopronin group (*p* = 0.349) suggesting that both groups were approaching the US normative mean in this domain. Role physical (tiopronin 47.2, non-tiopronin 43.9; *p* < 0.001), bodily pain (tiopronin 46.3, non-tiopronin 42.3; *p* < 0.001), general health (tiopronin 45.1, non-tiopronin 42.3; *p* < 0.001), vitality (tiopronin 48.2, non-tiopronin 45.3; *p* < 0.001), social functioning (tiopronin 47.6, non-tiopronin 43.9; *p* < 0.001), role emotional (tiopronin 47.7, non-tiopronin 45.3; *p* < 0.001) were all higher for the tiopronin group. The greatest differences in SF-36v2 HRQOL domain scores for the tiopronin group compared with the non-tiopronin group were in bodily pain, general health, and vitality.

Composite SF-36v2 scores were also higher for the tiopronin group. Physical component score (PCS) was slightly higher for the tiopronin group (49.5) compared with the non-tiopronin group (47.5, *p* < 0.001). Mental component score (MCS) was higher for the tiopronin group (46.9) compared with the non-tiopronin group (44.4, *p* < 0.001). No difference in the physical functioning domain between the tiopronin and non-tiopronin groups resulted in a smaller PCS difference than the MCS difference for the tiopronin group in the composite scores.

#### WISQOL scores

**Fig. 2:**
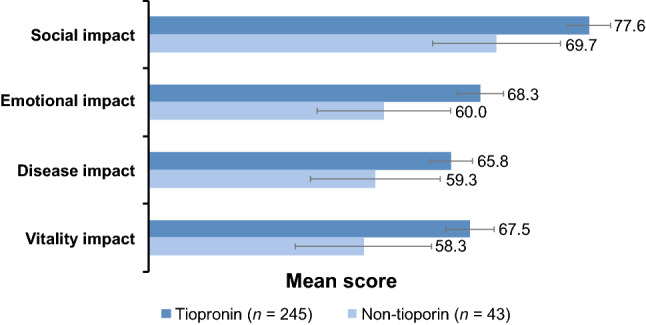
Mean WISQOL domain scores by treatment group. Error bars indicate standard error of the mean. *p* < 0.001 for tiopronin vs non-tiopronin groups (ANOVA) for each domain when controlled for time since the last stone event

The disease-specific WISQOL instrument measures four HRQOL domains: social impact, emotional impact, disease impact, and vitality impact [17]. Similar to the SF-36v2, it is important to control for the time between the last stone event and the survey. All the questions in WISQOL are asking about a participant’s quality of life for the previous 30 days. In this study, the tiopronin group had higher scores in all four HRQOL domains of the WISQOL instrument (*p* < 0.001): social impact (tiopronin group 77.6, non-tiopronin group 69.7), emotional impact (tiopronin 68.3, non-tiopronin 60.0), disease impact (tiopronin 65.8, non-tiopronin 59.3), vitality impact (tiopronin 67.5, non-tiopronin 58.3) (Fig. 2). The differences between the groups were similar for all four domains of the WISQOL instrument. The Levene test of variance revealed that variances were not equal in all domains.

Of note, the sample size for the WISQOL results was lower than the SF-36v2 results in this study, because the WISQOL questionnaires were not completed by some of the respondents.

#### Regression HRQOL analyses

In the eight domains of SF-36v2, the regression analyses indicated that the predictors explained 27.2–41.8% variance. Worse SF-36v2 scores were predicted by shorter time since last stone event for general health, social functioning, and role emotional. For physical functioning, role physical, and bodily pain domains, worse scores were predicted by shorter time since last stone event. For comorbidities, worse SF-36v2 scores were predicted in all domains with a great number of comorbidities (Table 3).

**Table 3:** Regression analyses for SF-36v2 scores in tiopronin-treated patients

	Physical functioning		Role physical		Bodily pain		General health		Vitality		Social functioning		Role emotional		Mental health	
	B	*p*	B	*p*	B	*p*	B	*p*	B	*p*	B	*p*	B	*p*	B	*p*
Age	− 0.13	0.001	− 0.061	0.141	− 0.029	0.474	0.06	0.154	0.08	0.046	0.03	0.440	0.03	0.501	0.11	0.005
Gender	− 2.391	0.034	− 3.83	0.002	− 2.46	0.045	− 3.082	0.008	− 3.61	0.002	− 5.37	0.00	− 4.71	0.00	− 3.65	0.002
Last stone event in groups	1.78	0.015	3.82	0.00	5.69	0.00	3.770	0.00	4.18	0.00	4.51	0.00	2.82	0.001	2.42	0.001
Comorbidities	− 2.26	0.00	− 2.58	0.00	− 2.23	0.00	− 3.007	0.00	− 2.682	0.00	− 2.90	0.00	− 2.36	0.00	− 2.55	0.000
*R*^2^	0.272		0.318		0.343		0.394		0.384		0.418		0.276		0.333	
Adjusted *R*^2^	0.259		0.306		0.331		0.382		0.373		0.407		0.262		0.320	
*F*	20.300		25.14		27.97		35.18		33.31		38.49		20.49		26.53	
Significance of *F*	0.00		0.00		0.00		0.00		0.00		0.00		0.00		0.00	
*N*	277		275		274		277		273		274		275		272	

Stone event groups: 1 = stone event within 30 days; 2 = stone event within 31–365 days, 3 = stone event > 1 year

*SF-36v2* short form-36, version 2

The results of regression analyses in the tiopronin group indicated that the predictors explained 36.4–38% of variance in domains of WISQOL. Worse WISQOL scores were predicted by shorter time since last stone event and more comorbidities. Gender also had a significant impact on each of the domain scores (Table 4).

**Table 4:** Regression analyses for WISQOL scores in tiopronin-treated patients

	Social impact		Emotional impact		Disease impact		Impact vitality	
	B	*p*	B	*p*	B	*p*	B	*p*
Age	0.12	0.252	0.14	0.239	0.09	0.36	0.071	0.546
Gender	− 12.08	0.00	− 13.06	0.00	− 10.749	0.001	− 13.25	0.00
Last stone event in groups	15.19	0.00	16.54	0.00	15.33	0.00	15.12	0.00
Comorbidities	− 4.865	0.00	− 4.693	0.00	− 4.93	0.00	− 6.35	0.00
*R*^2^	0.38		0.364		0.365		0.372	
Adjusted *R*^2^	0.368		0.351		0.352		0.360	
*F*	30.92		28.34		28.94		29.86	
Significance of *F*	0.00		0.00		0.00		0.00	
*N*	258		258		258		258	

Stone event groups: 1 = stone event within 30 days; 2 = stone event within 31–365 days; 3 = stone event > 1 year

*WISQOL* Wisconsin stone quality of life

#### Internal consistency

The Cronbach alpha coefficient was excellent for all four domain scores of WISQOL and was excellent (physical functioning, bodily pain, vitality, role emotional, and mental health) or good (role physical) or acceptable (general health) for domains scores of SF-36v2 (Table 5).

**Table 5:** Internal consistency in the overall population

HRQOL	Cronbach’s *α* coefficient	Single-measure intraclass correlation
SF-36v2		
Physical functioning	0.929	0.527
Role physical	0.899	0.663
Bodily pain	0.915	0.833
General health	0.768	0.411
Vitality	0.956	0.835
Social functioning	0.709	0.549
Role emotional	0.956	0.859
Mental health	0.992	0.960
WISQOL		
Impact on vitality	0.925	0.804
Disease impact	0.936	0.647
Social impact	0.947	0.686
Emotional impact	0.958	0.747

*HRQOL* health-related quality of life, *SF-36v2* short form-36, version 2, *WISQOL* Wisconsin stone quality of life

## Discussion

Due to the chronic and recurring nature of cystinuria, patient HRQOL can be significantly affected. Consistent with previous studies, the SF-36v2 and WISQOL scores in this study indicate that patients with cystinuria have impaired HRQOL [15, 16]. Even with a general quality of life measure, the SF-36v2, better HRQOL was detectable for the tiopronin group compared with the non-tiopronin group in bodily pain, general health, and vitality, when controlling for time since the last stone event [16].

The mean number of events, mean number of events requiring hospitalization, mean number of events requiring surgery, and mean number of extracorporeal shock wave lithotripsies were all higher in the tiopronin group, suggesting that the baseline characteristics for the tiopronin group contained a more severe cystinuria patient population than the non-tiopronin group. It is likely that patients were prescribed tiopronin as the result of a greater number of stone events. That domain scores were better in the tiopronin-treated patients is, therefore, quite noteworthy.

Statistical significance between the tiopronin and the non-tiopronin groups using ANOVA was observed in all the HRQOL domains included in the SF-36v2 and WISQOL instruments except for SF-36v2 physical function. The interpretation of SF-36v2 scores and differences in scores have led the developers of the instrument to propose minimally important differences (MID) for each of the domains in the SF-36v2: physical function, 3; role physical, 3; bodily pain, 3; general health, 2; vitality, 2; social functioning, 3; role emotional, 4; PCS, 2; and MCS, 3 [18]. Using this criterion, role physical (3.3), bodily pain (4.0), general health (2.8), vitality (2.9), social functioning (3.7), and PCS (2.0) were all above the MID threshold when comparing the tiopronin and non-tiopronin groups in this study. The MID findings further underscore the need to treat patients to help address the impact on HRQOL.

Limitations to the study exist. The response rate in the tiopronin group (24%) was greater than the non-tiopronin control group (17%), resulting in differences in the *n* values for the two groups (tiopronin, *n* = 267; non-tiopronin, *n* = 45). As these data were derived from mail-out surveys, it is difficult to determine why the response rate was different between the two groups, though the use of different registries for the tiopronin and non-tiopronin treatment arms may partially account for the difference. It is possible that patients enrolled in the Thiola^®^ Total Care Hub were more responsive than the non-tiopronin patients enrolled in the Cystinuria Contact Registry. Additionally, there were numerically more women than men in the non-tiopronin treatment group (78.8% vs 22.2%), whereas the tiopronin treatment group was more closely balanced by gender (46.1% vs 53.9%). The source of this difference in the control group is unclear but may be due in part to the comparatively limited sample size in the non-tiopronin group (*n* = 45).

It is also unclear if patients in the non-tiopronin group did not require tiopronin, have failed tiopronin therapy in the past, or did not want to take tiopronin. Prior tiopronin experience in the non-tiopronin population and length of exposure in the tiopronin-treated population were not captured in this survey. More than 95% of patients in the tiopronin group indicated that they were taking tiopronin as prescribed, while 46.8% of patients reported rarely missing tiopronin doses and 19.5% of patients reported missing doses on a daily basis. These findings are contradictory and there is some uncertainty in the adherence of tiopronin patients in this study, although adherence does generally appear high. Given the reported HRQOL for the tiopronin group, it is possible for even better HRQOL if there is a possibility for improvements in tiopronin adherence. Pak et al. describe that adherence with tiopronin leads to a nearly 70% disease remission rate [19]. If taking tiopronin per label leads to a decrease in stone recurrence, it can be assumed that those patients compliant with therapy may report improved HRQOL. Therefore, it is possible that the findings from this study are the result of a selection bias among those patients on tiopronin who responded to the survey.

Patients with cystine stones have been reported to have lower WISQOL scores specifically in areas related to sleep problems, nocturia, and feeling tired or fatigue [5]. When using SF-36v2, the cystinuria patients reported lower HRQOL than non-cystine stone formers in the role-emotional domain [16]. In this survey, the patients in the tiopronin group had better HRQOL than the non-tiopronin group for domains related to disease impact and emotional impact when assessed using both WISQOL and SF-36v2 questionnaire.

Overall, in cystinuria patients, tiopronin therapy resulted in better HRQOL compared with patients not on tiopronin treatment when assessed using the general questionnaire SF-36v2 and disease-specific questionnaire WISQOL. Our results support the therapeutic effect of tiopronin observed in a clinical study, where reduced rates of new stone formation and stone remission had been reported in cystinuria patients [12]. Additionally, the higher HRQOL demonstrated in the tiopronin-treated patients is an important consideration in this patient population where management often includes surgery, and HRQOL may not be a routine assessment.

## Conclusion

Compared with the non-tiopronin group, patients with cystinuria taking tiopronin reported better HRQOL as measured by WISQOL and SF-36v2. Healthcare professionals should consider how cystinuria may affect the HRQOL of their patients and offer measures that enhance treatment. Treatment with tiopronin may provide a positive effect on patients’ HRQOL concerns. Healthcare professionals should continue to monitor patients on tiopronin therapy and partner with patients on issues related to treatment management and disease control.

## Data Availability

My manuscript has no associated data or the data will not be deposited in a repository.

## Abbreviations

ANOVA: Analysis of variance
FDA: Food and drug administration
HIPAA: Health insurance portability and accountability act
HRQOL: Health-related quality of life
IRB: Institutional review board
MCS: Mental component score
MID: Minimally important differences
NYU: New York university
PCS: Physical component score
SF-36v2: Short form-36 version 2
WISQOL: Wisconsin stone quality of life questionnaire
